# 

**Published:** 1841-03

**Authors:** 


### CONTENTS

OF NO. III.

### ORIGINAL COMMUNICATIONS.

### ESSAYS AND CASES.

Art. I.—A Memoir on the Diseases called by the people "Trembles," and the "Sick-Stomach" or "Milk-Sickness;" as they occurred in the counties of Fayette, Madison, Clark, and Green in the State of Ohio. Read before the Medical Convention of Kentucky, Frankfort, January 12th, 1841. By Daniel Drake, M. D., of the Medical Institute of Louisville. - - - - 161

### SELECTIONS FROM AMERICAN AND FOREIGN JOURNALS.

Poisoning by Arsenic. - - - - - - - - 227

### ORIGINAL INTELLIGENCE.

Connecticut Retreat for the Insane. - - - - - - - 235
M'Clintock's Introductory Lecture. - - - - - - - 237
Professor Gross. - - - - - - - - - 239
Operation for Strabismus. - - - - - - - 240

